# ﻿ *Coptishuanjiangensis*, a new species of Ranunculaceae from Guangxi, China

**DOI:** 10.3897/phytokeys.213.96546

**Published:** 2022-11-15

**Authors:** Yiheng Wang, Jiahui Sun, Jingyi Wang, Qiang Mao, Wenpan Dong, Qingjun Yuan, Lanping Guo, Luqi Huang

**Affiliations:** 1 State Key Laboratory Breeding Base of Dao-di Herbs, National Resource Center for Chinese Materia Medica, China Academy of Chinese Medical Sciences, Beijing 100700, China China Academy of Chinese Medical Sciences Beijing China; 2 Key Laboratory of Biology and Cultivation of Herb Medicine, Ministry of Agriculture and Rural Affairs, Beijing 100700, China Ministry of Agriculture and Rural Affairs Beijing China; 3 Crop Research Institute, Sichuan Academy of Agricultural Sciences, Chengdu 610023, China Crop Research Institute, Sichuan Academy of Agricultural Sciences Chengdu China; 4 Laboratory of Systematic Evolution and Biogeography of Woody Plants, School of Ecology and Nature Conservation, Beijing Forestry University, Beijing 100083, China Beijing Forestry University Beijing China

**Keywords:** China, *Coptis*, Guangxi, new taxa, taxonomy

## Abstract

*Coptishuanjiangensis*, a new species of Ranunculaceae distributed in the valleys of Jiuwanshan National Natural Reserve in Huanjiang county (Guangxi, China), is described and illustrated for the first time based on morphological and plastome sequences data. It differs from *C.chinensis*, *C.deltoidei* and *C.omeiensis* mainly by having notably longer petiole, scape, bigger leaf blade with lobes obviously remote and robust rhizomes without stolons. Phylogenetic analyses support that *C.huanjiangensis* is sister to *C.omeiensis* and *C.deltoidei*.

## ﻿Introduction

The genus *Coptis* Salisb. (Ranunculaceae), containing 15 recognized species, is one of the most medicinally important genera in China and demonstrates a classical eastern Asian and North American disjunct distributional pattern. According to the morphology, especially flower and leaf, genus representatives distributed in China had been classified into six species and one variant, i. e., *C.chinensis* Franch. (endemic to SW China), C.chinensisvar.brevisepala W. T. Wang & P. K. Hsiao (endemic to SE China), *C.deltoidei* C. Y. Cheng & P. K. Hsiao (endemic to Sichuan, China), *C.omeiensis* (Chen) C. Y. Cheng (endemic to Sichuan, China), *C.quinquefolia* Miq. (distributed in Taiwan province and Japan), *C.quinquesecta* W. T. Wang (endemic to Yunnan, China) and *C.teeta* Wall. (endemic to SW China) (Tamura 1995; Dezhi and Robinson 2001).

All these species have branched rhizomes, basal and long petioled leaves splitting into three–five segments, small and actinomorphic flowers. Most of these species are less than 30 cm in height and grow in shady places in forest valleys at an altitude of 600–2500 meters. Due to the richness in benzylisoquinoline alkaloids, various *Coptis* species have been used in China ethnomedicine, and three of them, *C.chinensis*, *C.deltoidea*, and *C.teeta*, are used as official Huanglian ‘Weilian’, ‘Yalian’ and ‘Yunlian’ in the Chinese Pharmacopeia respectively (Liu et al. 2021; Wang et al. 2022).

The south-western limestone area is one of the biodiversity centers in China, especially in Guangxi (López‐Pujol et al. 2011; Huang et al. 2019). During the Fourth National Survey of Chinese Materia Medica Resources in Guangxi (August, 2018), we found an unusual species of *Coptis* with an outstanding plant size and robust rhizomes that are distinctive from other species in Jiuwanshan National Natural Reserve, Huanjiang county. Subsequently, an in-depth field investigation, detailed morphological observations and phylogenetic reconstruction by plastomes were carried out. The comprehensive morphology and molecular results suggested that it is a new species, which is described as follows.

## ﻿Materials and methods

### ﻿Taxon sampling and DNA extraction

Samples of the new species were collected in the field and 12 related species of *Coptis* (a total of nineteen accessions) were obtained from the herbarium of PE (Herbarium, Institute of Botany, CAS, Beijing, China) and CMMI (Institute of Chinese Materia Medica, China Academy of Chinese Medical Sciences, Beijing, China). *Asteropyrumpeltatum* and *A.cavaleriei* were taken as outgroups, and the plastome sequences were downloaded from GenBank (http://www.ncbi.nlm.nih.gov) with accession numbers MG734862.1 and MG734861.1, respectively. Sample information is listed in Suppl. material 1.

Total genomic DNA was extracted from specimens using a modified cetyl trimethyl ammonium bromide (CTAB) method and purified with the Genebetter DNA clean-up kit (GeneBetter Biotech Corporation, Beijing, China) (Li et al. 2013). All the DNA and molecular material were deposited in the herbarium of the Institute of Chinese Materia Medica (CMMI).

### ﻿Plastome sequencing and assembly

PE150 sequencing was conducted on an Illumina HiSeq XTen platform at Novogene (Tianjin, China). The raw data of the PE150 sequencing were filtered using the Trimmomatic 0.39 software to obtain high-quality reads (Bolger et al. 2014). The de novo assembly of the high-quality reads was performed by GetOrganelle v1.7.5 with the following settings: -F embplant_pt, -R 15 and -K 105 (Jin et al. 2020). Ambiguous regions and four junctions between IRs and SCs in the plastid were confirmed manually in Geneious v8.1 (Wang et al. 2021; Dong et al. 2022).

### ﻿Phylogenetic reconstruction

A total of 23 plastid sequences were aligned using the MAFFT online service and manually adjusted using MEGA X (Kumar et al. 2018; Katoh et al. 2019). And ambiguous regions were trimmed by the Gblocks 0.91b program (Castresana 2000). Phylogenetic reconstruction was carried out using the maximum likelihood (ML) and Bayesian inference (BI) methods in PhyloSuite (Zhang et al. 2020). The program ModelFinder was used to select the best-fit model according to the Bayesian information criterion (Kalyaanamoorthy et al. 2017). The ML tree was inferred using IQ-TREE with the TVM+F+R2 model and 5,000 ultrafast bootstraps (Nguyen et al. 2015). The BI tree was implemented with the GTR+F+I+G4 model and the Markov chain Monte Carlo (MCMC) chains were run for 1,000,000 generations. The trees were sampled every 1000 generations and the initial 25% were discarded as burn-in. Trees were visualized in FigTree v1.3.1.

## ﻿Results

### ﻿Phylogenetic analysis

**Figure 1.**
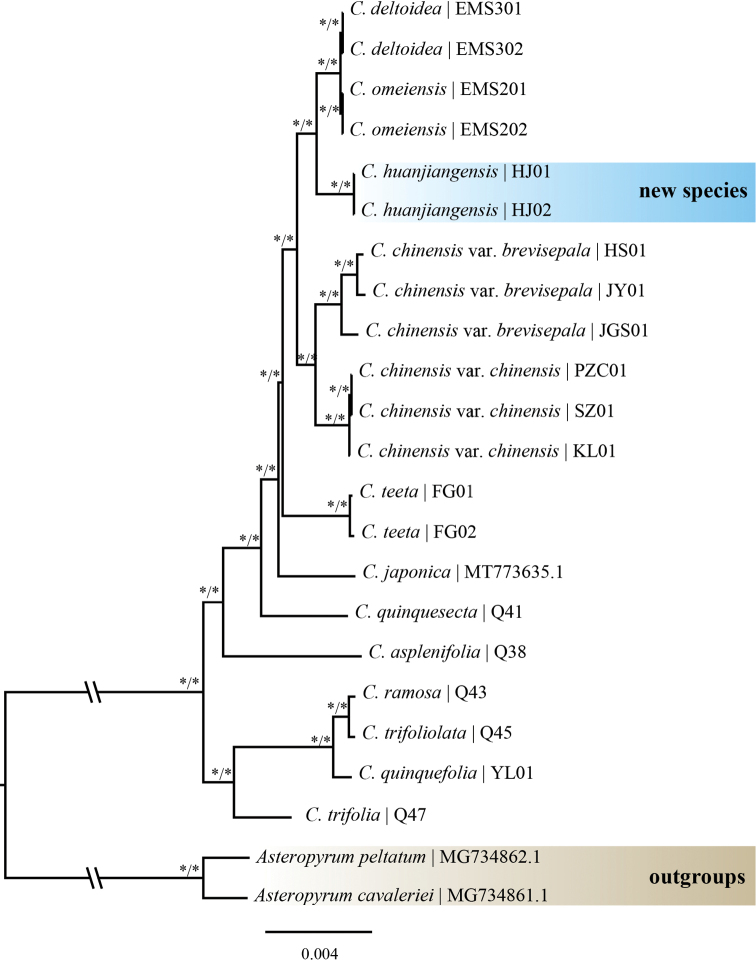
The Maximum likelihood and Bayesian inference tree of *Coptishuanjiangensis* and related species. Numbers on branches correspond to Maximum likelihood bootstrap support (BS) and Bayesian posterior probability (PP), respectively. An asterisk (*) indicates BS = 100% or PP = 1.0.

To better clarify the evolutionary position of the new species within *Coptis*, phylogenetic analyses were constructed based on the 23 complete plastid sequences with *Asteropyrum* as outgroups. The aligned sequences were 154,249 bp in length for analysis. The topologies of the ML and BI trees were identical with all the branches strongly-supported (ML BS = 100 and BI PP = 1) (Fig. 1). All the accessions of *Coptis* formed a monophyletic group with 100% support. The two samples of the new species (*C.huanjiangensis* sp. nov.) were clustered into one clade and sister to the clade consisting of *C.omeiensis* and *C.deltoidei*.

### ﻿Taxonomic treatment

#### ﻿ Coptis huanjiangensis

Taxon classificationPlantaeRanunculalesRanunculaceae

L.Q.Huang, Q.J.Yuan & Y.H.Wang sp. nov.

9E55CA0A-36A0-5B18-AD13-8B887F7FC1D3

urn:lsid:ipni.org:names:77308125-1

Figs 2 , 3

##### Diagnosis.

*Coptishuanjiangensis* is morphologically similar to *C.chinensis*, *C.deltoidei* and *C.omeiensis*, but it differs from these species by having notably longer petioles (15–40 cm), scapes (20–32 cm), and bigger leaf blades with lobes remote obviously.

##### Type.

China. Guangxi: Huanjiang County, Jiuwanshan National Natural Reserve, 1082 m, 25°12'1.07"N, 108°38'28.32"E, valleys, 24 January 2022, Yiheng Wang HJ220124I02 (holotype CMMI!, isotype CMMI!) (Suppl. materials 2, 3).

##### Description.

Herbs perennial, rhizomes branched, without stolons. Leaves basal, petiole 15–40 cm, glabrous. Leaf blade ovate-triangular, 12–22 × 9–22 cm, three-sect, papery to subleathery, abaxially glabrous, adaxially nearly glabrous on veins, base cordate, margin with sparsely upturned spiny hairs; central segment petiolulate (petiole 2.5–4 cm), ovate-rhombic, 11–18 × 7–14 cm, deeply four-ten-lobed, lobes remote, ultimate lobes margin acute serrate, apex acute or obtuse; lateral segments similar to or slightly shorter than the central one, obliquely ovate, unequally two-parted. Scapes one to several, erect, longer or shorter than the leaves, 20–32 cm tall, glabrous, sulcate. Inflorescences terminal, often monochasial, five-ten-flowered; flowers small, actinomorphic, bisexual; bracts lanceolate, palmately divided. Sepals five or six, greenish or redish yellow, long ellipsoid or lanceolate, 5.5–9.0 × 1.8–3.5 mm, sparsely puberulous. Petals spatulate, 2–5 mm long, glabrous, apex rounded to obtuse, 1/3–1/2 as long as sepals. Stamens numerous, glabrous, 2–4 mm-long, outer ones slightly shorter than petals. Pistils 8–14, 3–5 mm long; follicles 4.5–9.0 mm long, stipitate; seeds ellipsoid, ca. 1–2 mm long, brown.

**Figure 2.**
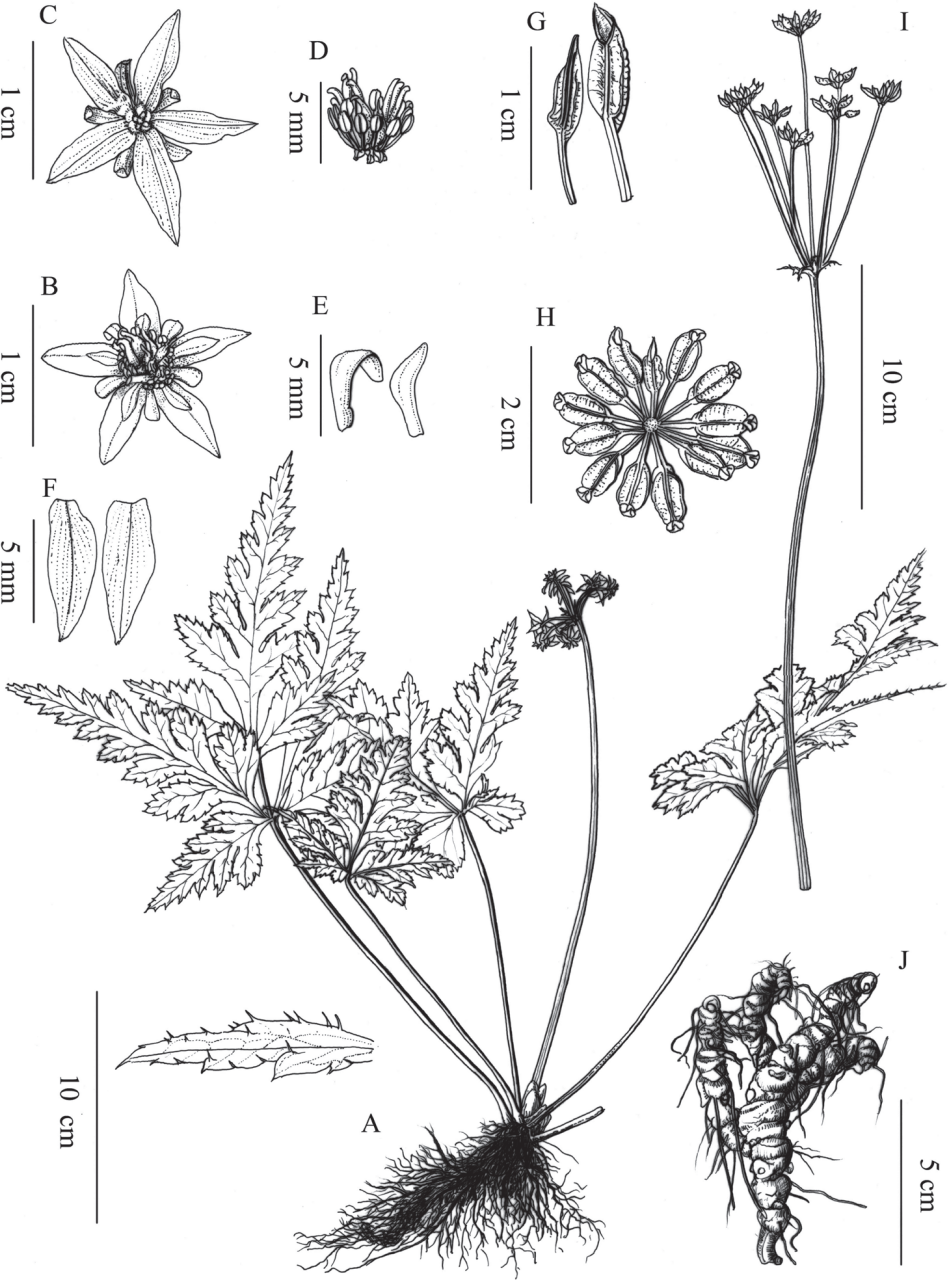
*Coptishuanjiangensis* L.Q.Huang, Q.J.Yuan & Y.H.Wang, sp. nov. **A** habit **B** flower, frontal view **C** flower, back view **D** opened corolla **E** petals **F** sepals **G, H** follicles **I** inflorescence **J** root. Drawn by Yingbao Sun.

**Figure 3.**
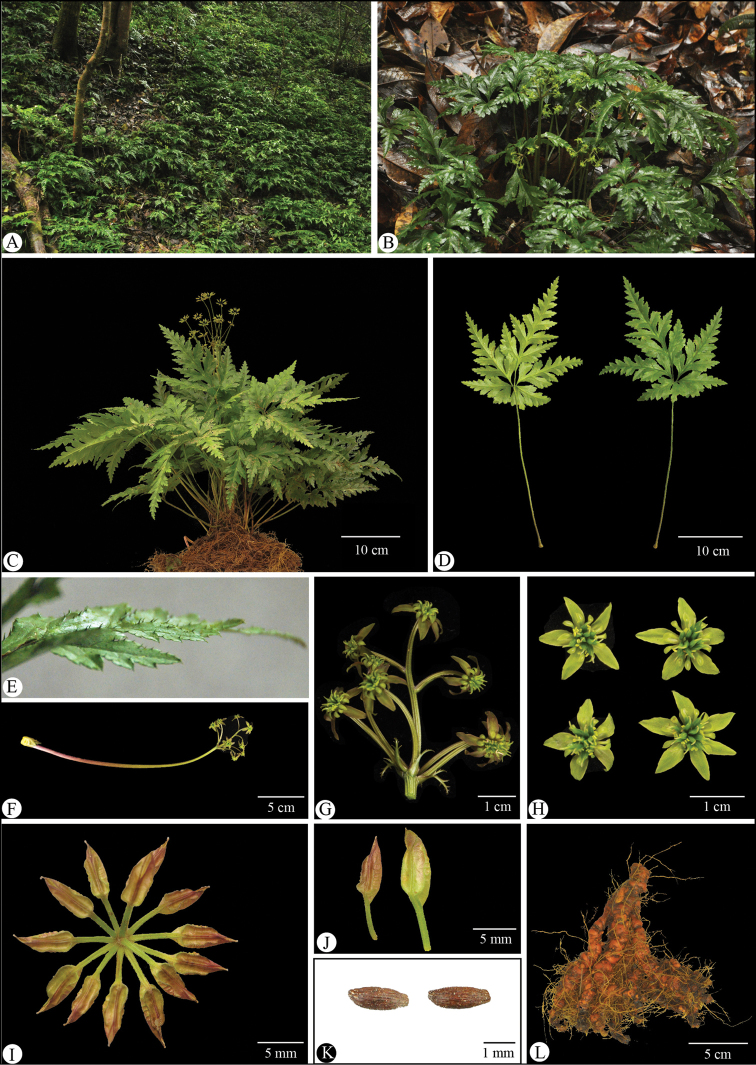
*Coptishuanjiangensis* L.Q.Huang, Q.J.Yuan & Y.H.Wang, sp. nov. **A** species habitat (Jiuwanshan National Natural Reserve, Huanjiang County, Guangxi, China) **B** plant in florescence stage **C** plant in fruiting stage **D** leaf, frontal and back view **E** margin with sparsely upturned spiny hairs **F–H** inflorescence and flowers **I–K** follicles and seeds **L** root. Photos by Yiheng Wang, Jingyi Wang & Qiang Mao.

##### Distribution and habitat.

This species has only been found in the valleys of Jiuwanshan National Natural Reserve, Huanjiang County up until now. It grows in shaded places in valleys at 800–1200 m. a. s. l.

##### Etymology.

The specific epithet is derived from the type locality, Huanjiang County, Guangxi.

##### Phenology.

The species was observed flowering in February – March and fruiting in April–June.

##### Note.

There are seven species and one variant of *Coptis* distributed in China. An identification key is presented below.

### ﻿Key to the species of *Coptis* in China

**Table:** 

1	Leaves five-sect	**2**
–	Leaves three-sect	**3**
2	Rhizome robust; leaf blade 5.5–14 cm wide, central segment pinnately divided, apex Attenuate	***C.quinquesecta***
–	Rhizome slender; leaf blade 2–6 cm wide, central segment three-lobed, apex acute	***C.quinquefolia***
3	Leaf blade lanceolate to narrowly ovate; lateral segments 3–3.5 × shorter than central segment; sepals linear-lanceolate	***C.omeiensis***
–	Leaf blade ovate to ovate-triangular; lateral segments slightly shorter than central segment; sepals lanceolate, elliptic, or narrowly ovate	**4**
4	Petals spatulate	**5**
–	Petals lanceolate to linear-lanceolate	**6**
5	Inflorescences three–five-flowered	***C.teeta***
–	Inflorescences more than five-flowered	***C.huanjiangensis***
6	Leaf segment lobes ± contiguous to each other; stamens ca. 1/2 as long as petals	***C.deltoidei***
–	Leaf segment lobes remote; outer stamens slightly shorter than petals	**7**
7	Sepals 9–13 mm, ca. 2 × as long as petals	**C.chinensisvar.chinensis**
–	Sepals ca. 6.5 mm, slightly longer than petals	**C.chinensisvar.brevisepala**

## ﻿Discussion

Plastoms have been extensively used in phylogeny reconstruction and species delimitation studies because of their moderate evolution rate and abundant phylogenetic information (Dong et al. 2021; Wang et al. 2021; Wang et al. 2022). The relationships of *Coptis* species were clearly resolved by phylogenetic studies. *Coptishuanjiangensis* possesses an independent phylogenetic position and is located in the clade formed by *C.chinensis*, *C.deltoidei*, and *C.omeiensis*. And the phylogenetic relationship of these four species is also supported by the morphological characters of these species in having a similar leaf blade shape (leaves three-sects), leaf blade texture (papery to subleathery), and a long and erect scape with five to ten small actinomorphic flowers. However, *C.huanjiangensis* can be distinguished from the latter three species by having notably longer petioles (15–40 cm) (vs other species having petioles shorter than 18 cm), spatulate petals (vs lanceolate or linear in other species), bigger leaf blades with lobes obviously remote and robust rhizomes without stolons. The detailed comparison between *C.huanjiangensis* and close species is represented in Table 1. Herein, both morphological and molecular studies indicated that *C.huanjiangensis* is an independent species.

**Table 1.** Distinguishing features of *C.huanjiangensis* in comparison with other related species.

Characters	*C.huanjiangensis*	*C.deltoidei*	*C.omeiensis*	C.chinensisvar.chinensis	C.chinensisvar.brevisepala
**Leaf blade**	ovate-triangular, 12–22 × 9–22 cm, papery to subleathery	ovate, 4–16 × 5–15 cm, papery to subleathery	lanceolate to narrowly ovate, 6–16 × 3.5–6.3 cm, subleathery	ovate-triangular, 4–10 × 4–10 cm, papery to subleathery	ovate-triangular, 4–10 × 4–10 cm, papery to subleathery
**Leaf margin**	deeply 4–10 lobed, lobes remote	4–6 lobed, lobes ± contiguous to each other	7–14 lobed, lobes remote	deeply 3–5 lobed	deeply 3–5 lobed
**Relationship of lateral segment and central segment in length**	lateral segments similar to or slightly shorter than central one	lateral segments slightly shorter than central one	lateral segments 3–3.5 × shorter than central one	lateral segments slightly shorter than central one	lateral segments slightly shorter than central one
**Petiole length**	15–40 cm	6–18 cm	5–14 cm	5–12 cm	5–12 cm
**Scape length**	20–32 cm	slightly longer than leaves	15–27 cm	12–25 cm	12–25 cm
**Sepal number**	5 or 6	5	5	5	5
**Sepal shape**	long ellipsoid or lanceolate	narrowly ovate	linear-lanceolate	lanceolate	lanceolate
**Petal shape**	Spatulate	lanceolate	linear-lanceolate	linear-lanceolate	linear-lanceolate
**The length ratio of sepal vs petal**	ca. 2–3 times	ca. 2–3 times	ca. 2 times	ca. 2 times	sepal slightly longer than petals
**Are there any stolons**	No	Yes	Yes	No	No

## Supplementary Material

XML Treatment for Coptis huanjiangensis

## Supplementary materials

Supplementary material 1 Accession number of 23 sequenced or downloaded chloroplast genome in this study Data type table (excel file) https://binary.pensoft.net/file/769795This dataset is made available under the Open Database License (http://opendatacommons.org/licenses/odbl/1.0/). The Open Database License (ODbL) is a license agreement intended to allow users to freely share, modify, and use this Dataset while maintaining this same freedom for others, provided that the original source and author(s) are credited.Yiheng Wang

Supplementary material 2 The photo of holotype stored at the CMMI with accession number HJ220124I02 Data type figure (JPG image) https://binary.pensoft.net/file/769796This dataset is made available under the Open Database License (http://opendatacommons.org/licenses/odbl/1.0/). The Open Database License (ODbL) is a license agreement intended to allow users to freely share, modify, and use this Dataset while maintaining this same freedom for others, provided that the original source and author(s) are credited.Yiheng Wang, Jiahui Sun, Jingyi Wang, Qiang Mao, Wenpan Dong4, Qingjun Yuan, Lanping Guo, Luqi Huang

Supplementary material 3 The photo of isotype stored at the CMMI with accession number HJ220124I01 Data type figure (JPG image) https://binary.pensoft.net/file/769797This dataset is made available under the Open Database License (http://opendatacommons.org/licenses/odbl/1.0/). The Open Database License (ODbL) is a license agreement intended to allow users to freely share, modify, and use this Dataset while maintaining this same freedom for others, provided that the original source and author(s) are credited.Yiheng Wang, Jiahui Sun, Jingyi Wang, Qiang Mao, Wenpan Dong4, Qingjun Yuan, Lanping Guo, Luqi Huang
