# Supplementary figures and images for: ﻿ Coptishuanjiangensis, a new species of Ranunculaceae from Guangxi, China
Source: PhytoKeys. 2022 Nov 15;213:131–41. doi: 10.3897/phytokeys.213.96546 (PMC9836509; doi:10.3897/phytokeys.213.96546)

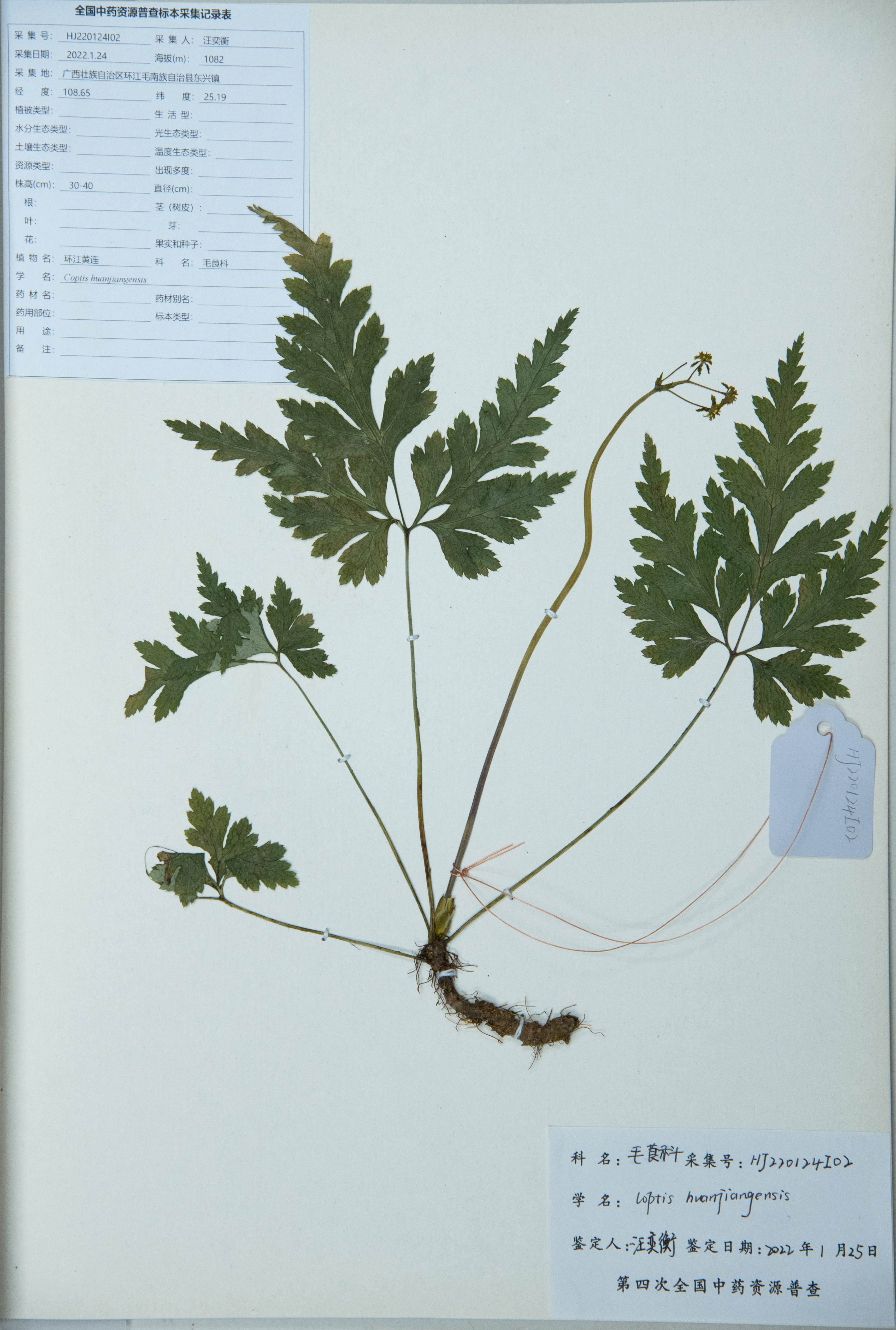

Supplement: Supplementary material 2 — The photo of holotype stored at the CMMI with accession number HJ220124I02 [file phytokeys-213-131_article-96546__-s002.jpg]

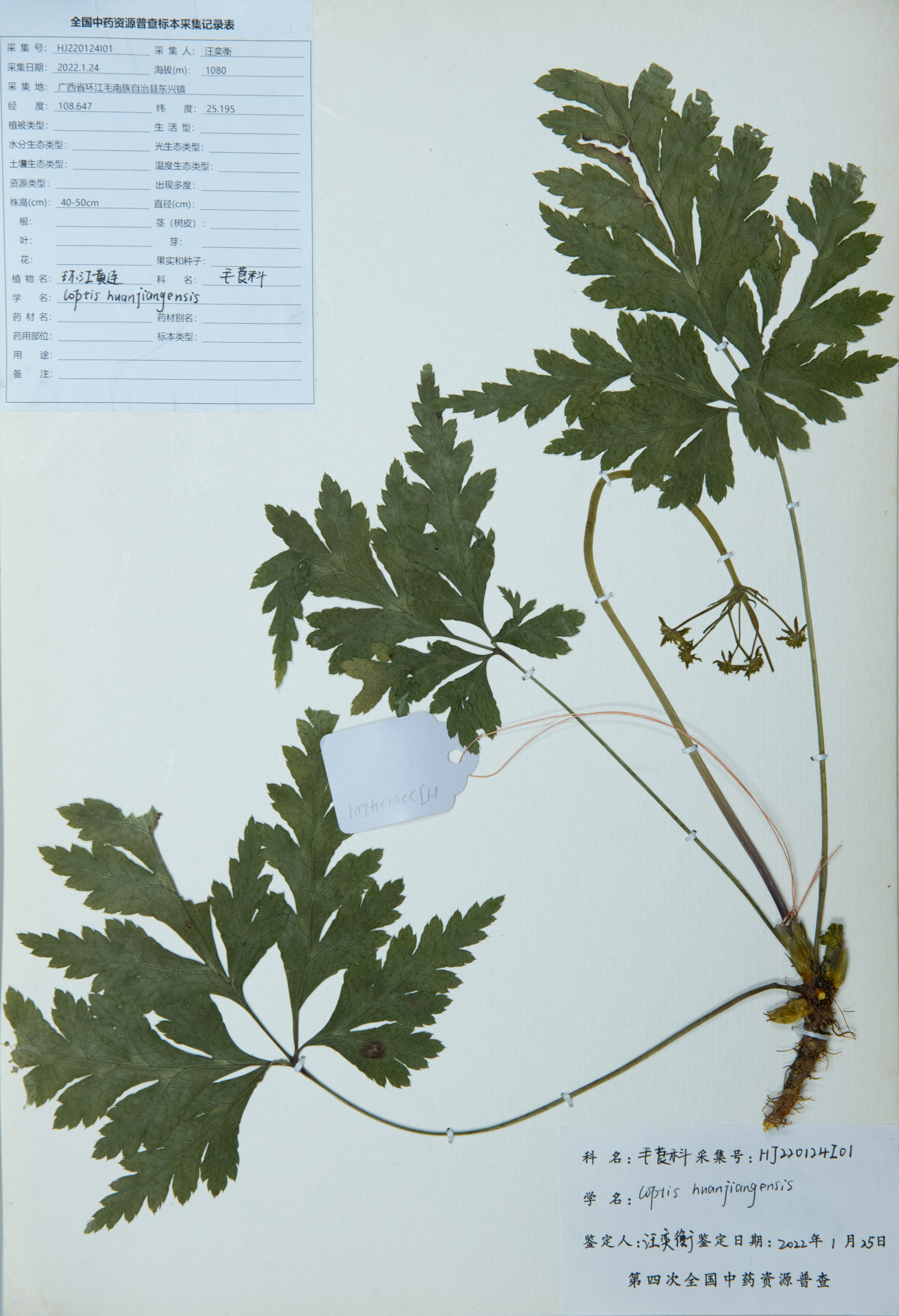

Supplement: Supplementary material 3 — The photo of isotype stored at the CMMI with accession number HJ220124I01 [file phytokeys-213-131_article-96546__-s003.jpg]
